# European violence risk and mental disorders (EU-VIORMED): a multi-centre prospective cohort study protocol

**DOI:** 10.1186/s12888-019-2379-x

**Published:** 2019-12-19

**Authors:** Giovanni de Girolamo, Giuseppe Carrà, Heiner Fangerau, Clarissa Ferrari, Pawel Gosek, Janusz Heitzman, Hans Salize, Margaret Walker, Johannes Wancata, Marco Picchioni

**Affiliations:** 1 Unit of Epidemiological and Evaluation Psychiatry, IRCCS Istituto Centro San Giovanni di Dio Fatebenefratelli, Brescia, Italy; 2 Department of Medicine and Surgery, University of Milano Bicocca (I), Milan, Italy; 3 Department of the History, Philosophy and Ethics of Medicine, Medical Faculty, Heinrich-Heine-University, Duesseldorf, Germany; 4 Unit of Statistics, IRCCS Istituto Centro San Giovanni di Dio Fatebenefratelli, Brescia, Italy; 5 Department of Forensic Psychiatry, Institute of Psychiatry and Neurology, Warsaw, Poland; 6 Central Institute of Mental Health, Medical Faculty Mannheim / Heidelberg University, Mannheim, Germany; 7 EUFAMI, Leuven, Belgium; 8 Clinical Division of Social Psychiatry, Department of Psychiatry and Psychotherapy, Medical University of Vienna, Vienna, Austria; 9 Consultant Forensic Psychiatrist, St Magnus Hospital, Surrey, UK; 10 Department of Forensic and Neurodevelopmental Science, Institute of Psychiatry, Psychology and Neuroscience, King’s College London, London, UK

**Keywords:** Forensic psychiatry, Violence, Risk assessment, Schizophrenia, HCR-20, Social cognition

## Abstract

**Background:**

The link between schizophrenia spectrum disorders (SSD) and violence is a core issue for most forensic psychiatric services. However, the drivers of violence in this population remain unclear, and, to date tools to predict violence risk have a range of limitations. Perhaps because of this uncertainty about the nature of violence risk, treatment programmes and care pathways for mentally disordered offenders vary substantially across the European Union, and differences in legal and policy frameworks are highly relevant.

**Methods:**

The three-year EU-VIORMED project (Grant Number PP-2-3-2016, November 2017–October 2020) involves forensic centres in Italy, Austria, Germany, Poland, and the U.K. It aims to: (a) identify and compare violence risk factors, clinical needs, and decision making capacity in violent (*N* = 200, “cases”) and nonviolent patients with SSD (*N* = 200; “controls”) using a case-control design; (b) test the predictive validity of the HCR-20v3, OxMIS and FoVOx among cases alone (*N* = 200), using a prospective cohort study; and (c) compare forensic-psychiatric care pathways across the EU, in a continent wide service mapping study.

**Discussion:**

Data collection started in September 2018 and continues. By September 2019, 333 participants have been enrolled (201 cases and 132 controls were recruited). Experts from 23 countries provided data for the service mapping exercise.

**Trial registration:**

Retrospectively registered on January 2, 2019 as researchregistry4604 January 2, 2019

## Background

Recent meta-analyses have demonstrated that schizophrenia spectrum disorders (SSD) are associated with a heightened risk of violent offending [1]: some studies have estimated that up to 20% of patients with schizophrenia in the community will behave violently in a six-month period [2]. Among violent offenders with SSD, there may be different subtypes [3]. One shows a pattern of antisocial behaviour which emerges in childhood or early adolescence, and remains stable across the life span; a second shows no antisocial features prior to the onset of psychosis, but exhibits frequent aggressive behaviour once the illness develops; the third is represented by a small proportion of SSD patients, does not show aggressive behaviour for a long time after the disorder begins, but then is associated with serious violence often towards caregivers [3].

One of the principle challenges of working in this field is the considerable variability of risk factors for violence amongst patients with SSD. The most robust risk factors for violence in patients with SSDs include: substance use disorders [4–6], poor insight, impulsivity, psychopathy, and cognitive abnormalities [5]; stressful life events [7]; family history of violence and child abuse [8, 9]; past violent behaviour [8, 10]; personal factors, such as male sex, age, lower intelligence, being single, a history of head trauma or neurological impairment, unemployment [9, 11, 12]; positive psychotic symptoms, such as persecutory delusions [2, 13], non-adherence to treatment [14], parental drug abuse [7] and childhood conduct disorder and victimization [2].

### Social cognition and risk of violence in people with SSD

The evidence for a link between neuropsychological deficits and violence in schizophrenia is very inconsistent [15, 16]. One recent study has suggested that the relationship between cognition and violence is largely mediated by social cognition deficits that may be independent of symptom severity [17]. However, studies on the role of empathy, that includes emotion recognition (ER) and Theory of Mind (ToM), and the risk of violence in people with SSD are inconsistent [18]. In a study by Demirbuga, Sahin [19], violent patients with SSD were found not to be impaired in facial ER compared to controls, both in terms of responses time and accuracy, while in another investigation violent patients with schizophrenia performed less well than healthy controls on ToM and ER tasks, but outperformed their controls with schizophrenia and no history of violence [20]. Furthermore, it has been suggested that there is a relationship between violence and hostile and externalizing attributional biases, whereby individuals with severe mental disorders attribute hostile intent to others are then more likely to behave violently [10, 21].

### Patients’ needs and treatment of offenders with SSDs

Since the 1990s there has been a growing consensus that mental health services must be much receptive and responsive to patients’ needs [22–25]. The majority of the existing literature assessing the needs of people with schizophrenia has focused on ‘civil’ patients with no history of violence [24, 26–28]. The needs’ assessment of forensic patients is comparatively underdeveloped [22, 24, 27, 29]: it may be that this is because forensic mental health services are still primarily focused on the assessment and management of violence risk and criminogenic needs [27, 28, 30, 31]. However, studies done so far have tended to find that both offenders without mental health problems and forensic patients have significantly more unmet needs than other groups [32–34].

### Ethical issues and patients’ competence

While political and societal ethical concerns in forensic psychiatry are mainly related to the stigmatization of forensic patients, clinical practice has to face four principle ethical dilemmas: respect for autonomy, beneficence, non-maleficence and justice [35], issues all linked to forensic patients’ competence, also known as capacity, to consent to their treatment. An in-depth assessment of forensic patients’ competence has never been undertaken using robust standardized methods. So far, the decisional capacity of forensic patients with psychotic disorders has been reported in only three studies [36–38], all from the same Irish research group, with relatively modest sample sizes and no comparison group. Hence, although they reported scores on the leading standardized tool (MacArthur Competence Tool) for their subjects, it was impossible to determine whether forensic patients were particularly impaired compared to non-forensic samples. Moreover, for those patients who lacked capacity to make decisions about their treatment, it was unclear whether and how their lack of capacity then affected the delivery of that treatment.

### Tools to predict and estimate the risk of violent behaviour

There are about 150 tools that claim to support violence risk assessment in psychiatric patients, though the evidence supporting their validity in patients with SSD is limited [39], and some authors have argued that their false positive rates are unacceptably high [40]. Structured professional judgement guides have a number of other limitations that include that they are often time consuming to complete, they have low predictive validity, lack specificity, rely on static factors, and need extensive rater training [39, 41, 42]. For these reasons most of these tools have failed to gain widespread acceptance in many clinical and forensic settings internationally. Furthermore while assessing and managing the risk of violence against other people is a core function of forensic services, it remains of critical importance to recognize and manage the significant risk of suicide in schizophrenia [43].

### Forensic care in Europe

While mental health care has seen marked changes in the last three decades, with a significant move from long-term hospital placement to community care, this has not been mirrored in most forensic settings. Generally speaking, between 1990 and 2006 the number of forensic beds more than doubled in some European countries, including Austria, Denmark, England, Germany, Ireland, the Netherlands, Spain and Switzerland [44, 45]. In a few countries, like Italy [46, 47], the number of forensic beds has decreased. The overall result is that forensic bed provision is very uneven across the continent. Furthermore, length of stay has increased in many countries, in part linked to societal demands for robust often coercive measures to be deployed against “dangerous” mentally disordered offenders with an ever-increasing focus on risk, but also in part because community resources have not developed to optimally support these people to live safely in the community. Detention and treatment in forensic settings may have become uncomfortably skewed towards public protection [48–50] rather than least restrictive patient care.

The increased demand for forensic services and beds can have several important implications. Firstly, in some countries ordinary mental health services have to treat increasing numbers of patients who have offended or who have been discharged from specialized forensic services. Alternatively, where forensic services have increased, resources may have been diverted from ordinary mental health services. Finally, forensic patients with SSDs can be doubly stigmatized through both having a serious mental disorder, but also because they have committed a criminal offense [51]. These can then cause downstream problems, for example restricting housing and vocational opportunities even when these patients are largely ready for life in the community [52].

### Pathways for care in- and out- forensic psychiatric services across Europe

Pathways into and out of forensic psychiatric services involve a variety of systems and professionals that span adult mental health services, the criminal justice system that includes the police, prisons, courts, probation services and social services. Patient pathways through forensic services, between and sometimes even within European countries are typically very unclear and inconsistent. For example, in some jurisdictions patients cannot be detained in hospital for longer than their equivalent prison sentence, while in others detention can be indefinite. There is very little comparative data about how patients enter and progress through forensic inpatient services from admission to discharge.

### Differences in service design and provision

While many studies have described forensic psychiatric services in individual countries [53–55], there are very few international comparisons. These comparisons are crucial, in particular when it comes to informing the commissioning, designing and reorganizing of future services that can be seen as inefficient [56]. International comparisons stimulate national debates, and ultimately lead to opportunities to disseminate and share best practices, possibly then leading to legal and clinical harmonization can increase international cooperation [57].

### Aims of the EU-VIORMED

The three-year EU-VIORMED project has five main aims:

1. to identify violence risk factors in patients with SSD and a history of significant violence (cases) and in patients with SSD and no history of significant violence (controls) using a case-control design;
2. to compare the rates of needs and unmet needs between cases and controls and to assess and interpret cases and controls’ competence to consent to treatment;
3. to test the validity of three violence risk assessment guides, the Historical Clinical Risk Management-20, Version 3 (HCR-20 V3), Forensic Psychiatry and Violence tool (FoVOx) and Mental Illness and Suicide Tool (OxMIS) in patients with SSDs and a history of significant violence, the cases, using a prospective cohort study design; and
4. to compare forensic-psychiatric care pathways across EU, conducting a mapping study;
5. to conduct two systematic reviews of the evidence to support pharmacological and non-pharmacological treatments for violence in patients with SSD in forensic settings.

**Fig. 1:**
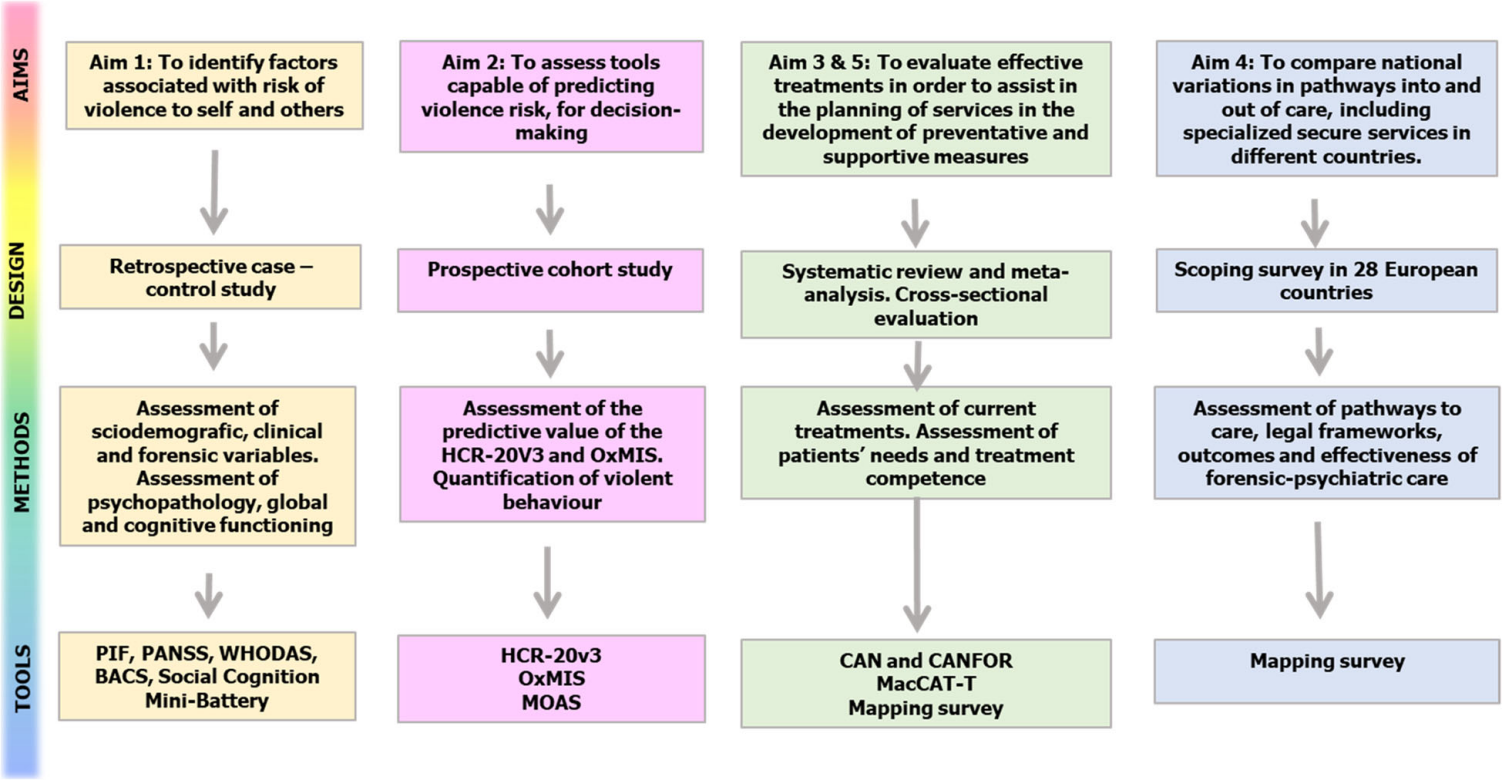
Overall design of the EU-VIORMED project

Figure 1 shows the main aims of the project and their flow along different steps.

## Methods/design

### Case-control study to identify violence risk factors and needs

**Fig. 2:**
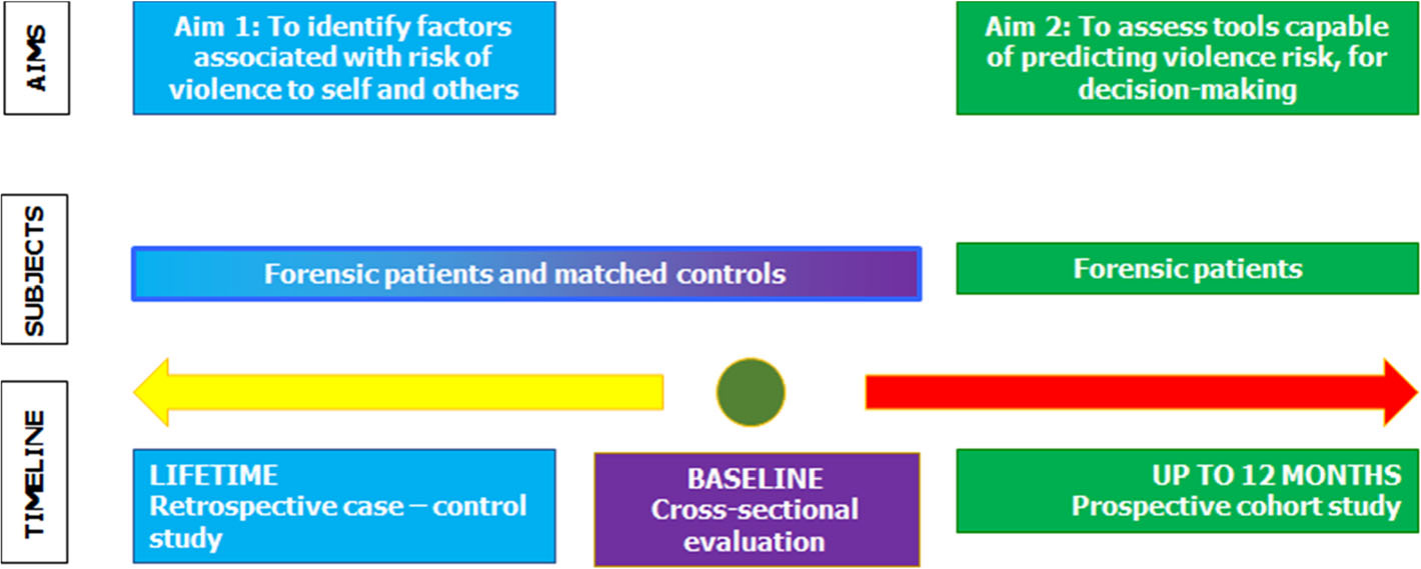
Overall design of the field work

The ‘core’ of the project is represented by a case-control study linked to a prospective cohort study, as shown in Fig. 2.

#### Hypotheses for the case-control study

**Fig. 3:**
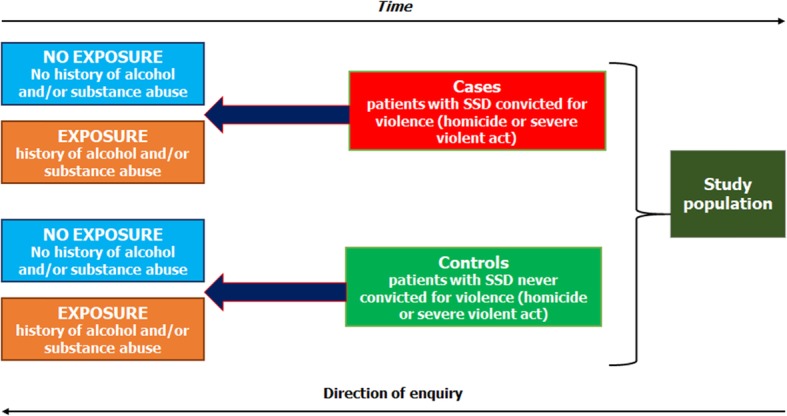
Model of a case-control design

In case-control studies, study groups are defined by outcome (Fig. 3) [58]. We explore each person’s exposure status to a range of putative risk factors using a retrospective study design.

In our case, we hypothesize that cases have experienced greater exposure to a range of violence risk factors that include substance misuse, domestic violence, and childhood trauma, and have experienced more severe positive psychotic symptoms compared to controls. We also hypothesize that violent cases would have a greater range of unmet needs than non-violent controls. Finally, given that most patients in a forensic setting are being held in those settings using some form of legal authority, we also hypothesized that forensic patients would show a lack of decision-making capacity compared to the control group.

#### Participants

Cases are defined as patients with SSD who have committed at least one act of serious violence (homicide, attempted homicide and violence causing serious physical damage) over the last 10 years and are recruited from forensic psychiatry settings. Controls are gender-and age matched patients with SSD who have never committed such acts of violence recruited from general adult settings.

Inclusion criteria for both groups were men and women of working age (18–65 years old) who met DSM-5 [59] criteria for a primary diagnosis of SSD. Exclusion criteria include: (a) a diagnosis of intellectual disability; (b) a diagnosis of a traumatic brain injury, cancer, organic brain disorders; (c) patients not able to speak the national language fluently; and (d) planned discharge in the next month.

All research subjects are reimbursed for their time, except in Italy because of ethical committee restrictions.

#### Settings

Additional file 1: Table S1 shows the different forensic and psychiatric facilities in the five countries where recruitment is taking place. They range from large forensic institutions in Germany and the UK, to smaller-scale (*N* = 20 beds) facilities typical of Italy. The EU-VIORMED consortium covers all four European regions (United Nations Statistics Division classification): Northern Europe (UK), Western Europe (Germany and Austria), Eastern Europe (Poland), and Southern Europe (Italy).

#### Procedure

Recruitment started in September 2018 and will be completed in September 2019 for cases, and March 2020 for controls. Controls are matched to recruited cases. In each study centre, treating clinicians invite patients with SSD to enter the study after reading written study information and having an opportunity to ask questions. All patients must indicate their consent to participate and to allow access to their medical records by signing.

#### Ethical permission

The project was approved by relevant local or national ethical committees of each country (Austria, England, Germany, Italy and Poland): the first approval was obtained by the St. John of God Ethical Committee (coordinating centre) on July 20th, 2018 (permission n. 74–2018); subsequent permissions have been obtained in each of the other recruiting countries according to national and local policies. The study is conducted in accordance with APA [60] guidelines and the Declaration of Helsinki [61].

#### Assessment instruments for sociodemographic and clinical variables

All patients are assessed through a set of standardized instruments within 3 weeks of recruitment. Table 1 shows the assessment instruments used by research assistants who received specific training in each instrument. Data is collected from patient and clinician interview and from examination of the medical records.

**Table 1:** List of assessment instruments for Eu-Viormed

Cases Forensic patients with SSD convicted for violence	Demographic and clinical data	*Patient Information Form (PIF)*
		*Analysis of patients’ forensic records*
	Violence risk factors	*Risk Factors Questionnaire*
	Psychopathology	*Positive and Negative Syndrome Scale (PANSS)*
	Assessment of health and disability	*World Health Organization Disability Assessment Schedule 2.0 (WHODAS)*
	Cognitive functioning	*Brief Assessment of Cognition in Schizophrenia (BACS)*
	Social Cognition	*Story-Based Empathy Task*
		*The Radboud Faces Database (RaFD)*
		*Individual decision-making tasks*
		*Cambridge Gambling Task (CGT)*
		*Moral Foundation Questionnaire (MFQ30)*
	Patients’ treatment needs	*The Camberwell Assessment of Needs Forensic version (CANFOR)*
	Decision making capacity	*MacArthur Competence Assessment Tool for Treatment (MacCAT-T)*
	Risk assessment	*Historical Clinical Risk Management-20, Version 3 (HCR-20 V3)*
		*Forensic Psychiatry and Violence tool (FoVOx) and Mental Illness and Suicide Tool (OxMIS)*
Controls Non-forensic patients with SSD never convicted for violence	Demographic and clinical data	*Patient Information Form (PIF)*
		*Analysis of patients’ forensic records*
	Psychopathology	*Positive and Negative Syndrome Scale (PANSS)*
	Assessment of health and disability	*World Health Organization Disability Assessment Schedule 2.0 (WHODAS)*
	Cognitive functioning	*Brief Assessment of Cognition in Schizophrenia (BACS)*
	Social Cognition	*Story-Based Empathy Task*
		*The Radboud Faces Database (RaFD)*
		*Individual decision-making tasks*
		*Cambridge Gambling Task (CGT)*
		*Moral Foundation Questionnaire (MFQ30)*
	Patients’ treatment needs	*Camberwell Assessment of Need (CAN)*
	Decision making capacity	*MacArthur Competence Assessment Tool for Treatment (MacCAT-T)*

Socio-demographic variables are assessed using a study specific ***Patient Information Form (PIF)*.** It collects socio-demographic data (i.e. age, gender, education level, socio-economic status), clinical data (i.e., age at disease onset, medications, adherence to medication, substance use disorders), and information about past offending (the patient’s mental state at time of violent offending, the pathways of care both before and after committing the crime, the consequences for the perpetrator and the victim, and the environmental context in which the violence took place).

The ***patients’ medical records*** are examined for entries that recorded consent and refusal decisions for treatment with medication or psychosocial treatments, situations where refusals were overridden, the reasons for overriding the patients’ refusal and process by which it was accomplished.

The ***Risk Factors Questionnaire***, a study specific form to collect data on violence risk factors in psychiatric patients, was developed based on previous data [62]. It is administered only to cases.

The ***Positive and Negative Syndrome Scale*** **(PANSS)** [63] is used to assess current psychopathology. The 30-items (rated from 1 to 7) allows the assessment of a multidimensional range of symptoms typical of schizophrenia, including positive and negative syndromes, and general severity of illness. The PANSS is scored on the basis of patient and caregiver reports, and clinical observations. It has good criterion-related validity and predictive validity [63].

The ***World Health Organization Disability Assessment Schedule 2.0*** **(WHODAS 2.0)** [64] is a 12-item measure, which includes 6 functional domains (i.e., cognition, mobility, self-care, getting along, life activities, participation). WHODAS 2.0 has good reliability and validity across a variety of clinical populations and settings [65, 66].

The ***Brief Assessment of Cognition in Schizophrenia*** (BACS) was specifically designed for use in schizophrenia clinical trials. It is brief portable and repeatable. It assesses cognitive domains that are consistently impaired and closely linked to outcomes in schizophrenia, that include verbal memory and learning, working memory, motor function, verbal fluency, processing speed and executive function. The BACS has been translated and validated in many different languages, and has shown excellent psychometric properties [67].

#### Assessment of social cognition

Due to security considerations, the social cognition assessment takes place only in Italy and Poland.

The ***Story-Based Empathy Task*** [68] is a non-verbal test for the assessment of intention and emotion attribution in clinical conditions. It includes 18 stimuli, sub-grouped into two experimental conditions assessing, respectively, the ability to infer others’ intentions and emotions compared to a control condition of causal inference.

The ***Radboud Faces Database*** (RaFD) [69] is used for emotion recognition, a subtype of social cognition. RaFD is a set of pictures of 67 models displaying 8 emotional expressions (i.e. joy, fear, disgust, sadness, surprise, anger, contempt and neutral). Participants are asked to identify the correct emotion. The task has good experimental properties [69].

Individual decision making is evaluated with two tasks which specifically assess: (a) loss aversion, i.e. the tendency to overweight the negative, compared with positive, consequences of choice [70]; (b) delay discounting, i.e. the tendency to discount delayed rewards [71].

Decision-making and risk-taking behaviour is evaluated with the ***Cambridge Gambling Task*** (CGT) [72]. The participant is presented with a row of ten boxes red or blue boxes. The ratio of red and blue boxes varies between stages, one box always contains a yellow token. Participants must use the ‘Red’ and ‘Blue’ buttons to choose the box color in which they think the yellow token is hidden. Outcome measures include measurements of risk taking, quality of decision-making, decision time, risk adjustment, delay aversion and delay aversion/impulsivity.

The ***Moral Foundation Questionnaire*** (MFQ30) [73] assesses the degree to which an individual’s moral beliefs and concerns rely upon different moral domains. According to the Moral Foundation Theory [74], the MFQ30 describes 16 situations that posit five foundations of morality constructs: Harm/Care, Fairness/Reciprocity, Ingroup/Loyalty, Authority/Respect, and Purity/Sanctity.

#### Needs’ assessment

The ***Camberwell Assessment of Need*** **(CAN)** [75] and the ***Camberwell Assessment of Needs Forensic version*** **(CANFOR)** [25] are used to assess the controls’ and cases’ needs respectively. The CAN assesses needs across 22 domains, classifying them as met or unmet, as well as the support and help offered in each domain. Domains include: accommodation, food, looking after home, self- care, daytime activities, physical health, psychotic symptoms, information about treatment and condition, psychological distress, safety to self, safety to others, alcohol, drugs, company, intimate relationships, sexual expression, childcare, basic education, telephone, transport, money and social benefits. The CAN is valid and reliable for people with severe mental disorders. The CANFOR is used for assessing needs among cases. It has the same format as the CAN, with three additional areas namely treatment, arson and sexual offending [76].

#### Assessment of patients’ competence to consent to for treatment

The ***MacArthur Competence Assessment Tool for Treatment*** (MacCAT-T) [77–79] is a semi structured instrument to assess decisional capacity in psychiatric patients. The instrument assesses patients’ competence to make treatment decisions by examining their abilities in four areas: understanding information relevant to their condition and treatment, reasoning about the potential risks and benefits of their choices, appreciating the nature of their situation and the consequences of their choices, and expressing a choice.

#### Statistical issues and sample size determination

Study group was defined as a dichotomous variable with the categories ‘cases’ and ‘controls’. The study sample size was calculated as the minimum number of subjects to obtain a significant risk factor exposure as measured by Odds Ratio (OR) from a logistic regression model. Based on data in Fazel, Buxrud [62], the lower effect size reported was OR = 2.2. Considering this effect size and by using a two-tailed logistic regression z-test for binomial distributed exposure variables with the following input parameters (in Gpower software):

i. the probability of an event under null hypothesis H0 [Pr(Y = 1|X = 1) H0] = 0.3 (evaluated as an average of the probabilities quoted in Fazel et al., 2010);
ii. power: between 0.85–0.90;
iii. significance alpha level = 0.05;
iv. prevalence of exposure (i.e. prevalence of the risk factor) of 0.5;

a sample size of *N* = 250: 125 controls and 125 cases was obtained. The sample size was then adjusted for the Design Effect (DE) [80] due to the multicentre nature of the study, defined as DE = 1 + (N/m-1) *ICC (where m is the number of sites, ICC is the intra-class correlation coefficient chosen equal to 0.05), leading to a sample size of *N* = 373. Finally, we predicted a drop-out of 10%, so the final sample size needed to detect a significant effect of the risk factors on violent outcome (cases vs controls) was deemed to be *N* = 400 (200 controls vs 200 cases, corresponding to 40 controls and 40 cases per country). Descriptive statistics will be calculated to examine demographic and clinical features. Appropriate statistical tests will be used to examine whether prevalence of violence, risk levels, and sample characteristics differ between national samples.

Almost all included patients have been able to score the comprehensive data battery. The rate of refusal has been much higher in three participating countries (Austria, England and Germany) as compared to other two (Italy and Poland).

### Prospective cohort study to assess the predictive value of risk violence tools

#### Hypotheses

We test the predictive validity of the HCR-20v3 [81], FoVOx [82] and OxMIS [83] over a 12-month follow-up period among the cases only. We hypothesize that FoVOx will predict the risk of future violence among cases as accurately as the HCR-20v3 over a 12-month follow-up period, and that OxMIS will reliably identify those at high risk of suicide.

#### Assessment instruments and primary outcome

The ***Historical, Clinical, Risk-20v3*** **(HCR-20v3)** [81] is a structured professional judgement guide administered to cases to evaluate their future violence risk. It rates the presence and relevance of twenty key violence risk factors, and leads to an overall summary judgement of the risk of future violence. It has been translated into many languages, and is widely used in various forensic and correctional settings.

The ***Forensic Psychiatry and Violence Tool*** **(FoVOx)** [82] is a web based actuarial violence prediction guide developed by Fazel and colleagues. It has 12-items and showed at 24 months post-discharge, using a 5% cut-off, sensitivity of 96% and specificity of 21%; positive and negative predictive values were 19 and 97%, respectively. Using a 20% cut-off, sensitivity was 55%, specificity 83% and the positive and negative predictive values were 37 and 91%, respectively.

The ***Mental Illness and Suicide Tool*** **(OxMIS)** [83] is a seventeen item web-based actuarial risk instrument for suicide. It generates a simple output – probability scores (%) based at 12-month risk of suicide.

The ***Modified Overt Aggression Scale*** **(MOAS)** [84] is used to code all violent incidents, over a 12 month follow-up period and is the primary end-point for the cohort study. The MOAS is rated based on staff report at 3, 6, 9 and 12 months after initial risk assessment rating. The MOAS includes four violence subdomains: verbal violence, violence against objects, violence against self, and physical-interpersonal violence. Each subdomain is rated from 0 to 4 according to the most serious incident over the follow up period: 0 indicates no violent behavior, higher scores indicate greater severity. The weighted MOAS score, the primary outcome in this part of the study is calculated by applying a factor weighting to each of the subdomains (1 for verbal violence, 2 for violence against objects, 3 for violence against self, and 4 for violence against other people). The total weighted score for each 3-month follow-up period thus ranges from 0 (no violence) to 40 (maximum across all subdomains).

#### Participants and procedure

**Fig. 4:**
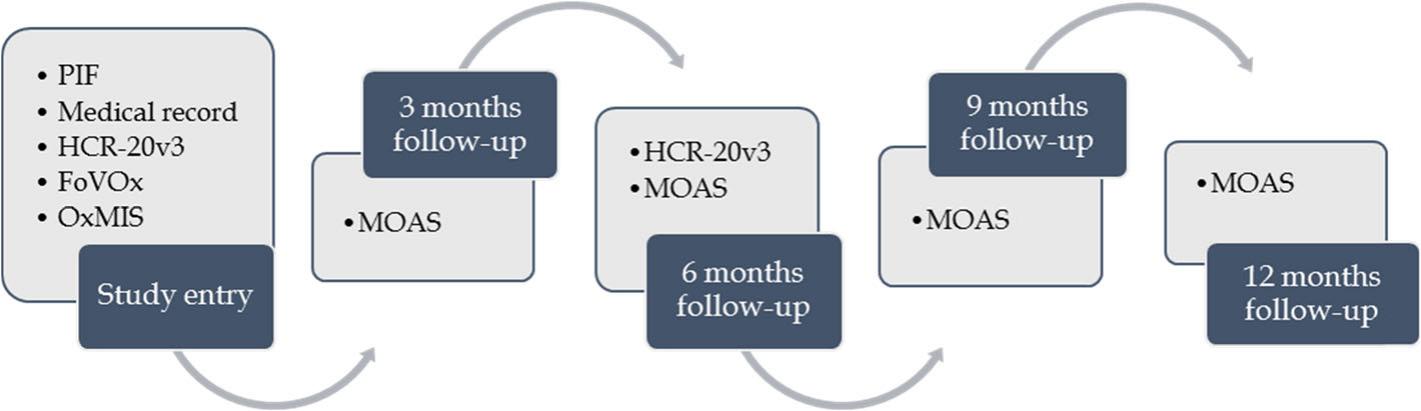
Summary of design and measures of study 2 (prospective cohort study)

Cases included in this study are the same cases identified in the case-control study described above. At study entry, cases are assessed for their future violence risk using the HCR-20v3, the FoVOx and the OxMIS, as shown in Fig. 4. The HCR-20v3 is rated again at 6-month follow-up, as this is the accepted upper limit of its window of validity. The MOAS is rated at months 3, 6, 9, and 12 after study entry to objectively record any violent incidents that occurred based on staff report. If a patient is discharged during the 1-year follow-up, attempts are made to rate the MOAS based on information received from the supervising clinician.

#### Statistical issues

The sample size was calculated for the prospective study to evaluate the violence risk predictive validity of the HCR-20 v3. Using the same Gpower software as above, and setting the exposure as a normal variable with parameters (mean = 20, standard deviation = 6, evaluated as an average of the value reported for the HCR-20v3), *N* = 200 is sufficient to detect a small effect size OR = 1.07 (published data on the OR for HCR-20 are greater than 1.2–1.3; [85]).

Differences in mean HCR-20v3, FoVOx, and OxMIS scores between national samples will be investigated using independent parametric (t test) or non-parametric (for non-Gaussian data) tests as well as linear (ANOVA) or generalized linear models. The predictive validity of the HCR-20v3, FoVOx and OxMIS scales will be assessed using the area under the curve (AUC) values generated from receiver operating characteristic (ROC) analyses and differences in their performance will be identified using the rocreg function in Stata, which calculate sensitivity and specificity while controlling for covariates.

#### Status

Data collection started on 1 September 2018 and continues successfully. By 30 September 2019, 201 cases and 132 controls have been enrolled, and 144 potentially eligible have declined.

### Mapping of forensic care in Europe

#### Aims

The aim of the European forensic service mapping is to compare the legal regulations and processes for mentally disordered offenders, the forensic service configurations and characteristics, and national variations in pathways into and out of care, across the EU member states.

#### Participants

The study has involved country specific experts within each of the twenty-eight EU member states. They were selected from formal or informal forensic psychiatric expert networks, such as the COST Action on Long-Term Forensic Psychiatric Care (IS 1302) and the Ghent-group. Some experts, in particular from countries lacking a stronger tradition in the field were included by recommendation from colleagues of neighboring or other countries. In some cases, refusals from experts of some countries required substitutes to be identified. By September 2019, twenty-one mapping questionnaires had been received.

#### Procedures

A study specific service mapping questionnaire was developed: it includes a mixture of quantitative and qualitative data that comprises sections on the prevalence of mentally disordered offenders, numbers of officially assigned forensic psychiatric beds, descriptions of basic legal concepts and frameworks for detention or treatment of mentally disordered offenders, court and trial procedures, and assessment or discharge practices or statutes; overall the forensic mapping questionnaire includes 10 sections, some with multiple sub questions. The data will be used for in-depth comparative analyses on the concepts, design, and treatment models that influence the treatment practices of mentally disordered offenders across all the EU states.

### Systematic reviews

#### Aims

There is a growing body of literature that examines pharmacological and non-pharmacological management of violence in people with SSDs, though few have focused on their use in forensic settings. We are thus conducting systematic reviews of the existing literature on the pharmacological and non-pharmacological management of violence in patients with SSDs living in forensic settings.

#### Procedures

Queries are limited to articles published since 1990 and reporting data on violence in adult forensic psychiatric patients, using pre-specified keywords. Only articles published in peer-reviewed journals are considered, in order to limit the search to studies with an adequate level of methodological rigor. We used 1990 as the starting point for the search in recognition of the different way mental health care was provided, and differences in the way adverse events might have been recorded in the past.

Study quality is assessed using a four-point “strength of reporting” scale, derived from the Strengthening the Reporting of Observational Studies in Epidemiology (STROBE) statement checklist [86]. Two reviewers independently review every study abstract to identify relevant reports. Randomized controlled trials are analysed separately. Whenever possible, intention-to-treat data is used. For binary outcomes, we calculate a standard estimation of the Risk Ratio (RR) and its 95% confidence interval (CI). For statistically significant results, we also calculate the number needed to treat to provide a benefit and its 95% CI. For continuous outcomes, we estimate the Mean Difference (MD) between groups. The possibility of statistical heterogeneity is investigated by visually inspection of graphs and by considering the I2 statistic [87]. We use the GRADE approach to interpret findings [88]. Results from these reviews will help identify the most effective treatments of violence in patients with SSDs in forensic settings and so assist managers, commissioners and clinicians with services planning.

## Discussion

People with SSDs who have been violent are a stigmatized and marginalized group, generally seen as difficult-to-manage and disruptive. The violence they are involved in whether as perpetrator or victim seems to have a profoundly detrimental effect on wider public opinion, leading to discrimination. This then increases further the burden on family members and care givers who are in fact most often the victims of that violence. The best way to support and treat such patients and their carers is evolving in European forensic mental health care systems, yet still represents a huge challenge for clinicians, the police and judiciary, commissioners and funders, as well as for carers and family and indeed for society at large.

Despite their large resources demands, while as a whole mental health services in many EU states have evolved over the last 25 years [89], forensic services in most European countries have experienced a comparatively slower rate of innovation and are embryonic in some countries. At the same time, while the European Commission has deemed mental health research a priority area, forensic psychiatry has done very poorly. The CORDIS archive of all European-funded projects shows that of 890 projects supported by the European Commission over the last 25 years, only six were in forensic psychiatry. This highlights the weakness of research in the field and the need to promote and strengthen collaborative research networks across Europe: EU-VIORMED is one of the first steps to achieve that target.

Balancing the paradox of treatment compulsion and informed consent, care and effective risk management are core tasks of forensic psychiatry. Forensic psychiatrists must deal with patients who are generally hospitalized against their will, often for long periods of time and with substantial restrictions imposed on them. Yet forensic psychiatry patients remain at their core ‘patients’, who deserve care and treatment. While these patients are often unable to consent to the treatments they need, or even actively refuse them, the principle of informed consent must remain a crucial element of the patient-physician relationship. Care and treatment must never be seen as a punishment, but in the forensic mental health setting coercive treatment is sometimes needed and the right thing to do.

Perhaps one way to ease this tension might therefore be to gain a greater understanding of patients’ views on services and their own clinical needs within the forensic care system. While it is important to identify what treatments are effective, it is also important what the patients think about treatment and what satisfaction with a service means to them. Previous research on forensic mental health services have largely ignored patients’ views about the nature and quality of services offered, underlining the immediate need for a comprehensive understanding of patients’ views on their needs across different forensic settings.

The EU-VIORMED project will help us understand better what causes people to be violent, how to more accurately assess their future risk, what treatments may work best to reduce the risk of violence, how services are currently structured and, perhaps most importantly, what patients feel a good service would look like. The aim is to provoke national and international debate on these issues through data to foster a better understanding of the clinical, ethical and personal issues to deliver better forensic psychiatric care across the EU.

With regard to winder national policy issues, the data from at least 23 European countries will allow firstly a meaningful comparison of forensic service configurations across the EU, and will be an initial driver to promote harmonization of treatment and perhaps legal pathways for people with SSD who have offended. This, in turn, may help to promote changes and reform in all forensic and penal systems.

An already emerging theme besides the need to strengthen clinical forensic mental health services is the clear need for better collaboration with other statutory agencies, such as housing, social care and the criminal justice system as well as third sector agencies and families.

### Limitations of the EU-VIORMED

While great efforts were made to find the best balance between scientific rigor and feasibility, the distinctively conservative nature of forensic services has imposed some limitations on patients’ assessment. For example, due to time constraints we did not include any specific structured assessment for personality disorders or trauma. We were also unable to include an assessment of treatment engagement and progress, such as the DUNDRUM for similar reasons and of physical health and activity parameters.

The study will assess the validity of the violence risk assessment guides while the patients remain under the care of forensic services, and thus while they remain subject to active risk management. This may be a confounder when looking at the follow-up violence and self-harm data.

We were also unable to include an assessment of carers’ needs and engagement, a clear limitation. Finally, despite our ultimate aim of promoting the homogenization of forensic practice across Europe, it must be noted that despite using clear definitions for cases, there are discrepancies in how clinical and legal systems operate that may cause differences between national forensic populations. For example, specific patient subgroups might be treated in forensic settings in one country, but specifically excluded in another.

## Conclusions

The EU-VIORMED study was designed to increase our understanding of personal and clinical factors associated with the risk of violence in patients with SSDs. It strives to expand and develop our understanding of violence risk assessment and consolidate our understanding about what works in terms of treatment and practice. Its ultimate aim is to help us develop and deliver more timely, effective, evidence-based and acceptable care. It aims to update our awareness of the broad range of service design and delivery models across the EU, and finally to provoke thought on the legal and ethical issues about the use of security, the law and enforced treatment in the EU in the twenty-first century.

## Supplementary information

**Additional file 1:** **Table S1.** List of recruiting forensic sites and heads.

## Data Availability

The project will fully embrace the open access data policy of H2020 to make data FAIR (Findable, Accessible, Interoperable, and Re-usable), and all data gathered in the framework of the project will be stored in a public repository, accessible to all scientists willing to carry out additional analyses.

## Abbreviations

BACS: Brief Assessment of Cognition in Schizophrenia
CAN: Camberwell Assessment of Need
CANFOR: Camberwell Assessment of Needs Forensic version
CGT: Cambridge Gambling Task
ER: Emotion Recognition
EU-VIORMED: European Violence Risk And Mental Disorders
FoVOx: Forensic Psychiatry and Violence tool
HCR-20 V3: Historical Clinical Risk Management-20, Version 3
MacCAT-T: MacArthur Competence Assessment Tool for Treatment
MFQ30: Moral Foundation Questionnaire
MOAS: Modified Overt Aggression Scale
OxMIS: Mental Illness and Suicide Tool
PANSS: Positive and Negative Syndrome Scale
PIF: Patient Information Form
RaFD: Radboud Faces Database
SSD: Schizophrenia Spectrum Disorders
ToM: Theory of Mind
WHODAS 2.0: World Health Organization Disability Assessment Schedule 2.0
