# Supplementary material for: European violence risk and mental disorders (EU-VIORMED): a multi-centre prospective cohort study protocol
Source: BMC Psychiatry. 2019 Dec 19;19:410. doi: 10.1186/s12888-019-2379-x (PMC6924026; doi:10.1186/s12888-019-2379-x)
Supplement: Supplementary file 1 — Additional file 1: Table S1. List of recruiting forensic sites and heads. [file 12888_2019_2379_MOESM1_ESM.docx]

Table S1 List of recruiting forensic sites and heads

| COUNTRY | TYPE OF FACILITY OR SERVICE | NUMBER OF FORENSIC BEDS* | FACILITY NAME AND LOCATION | HEAD |
| --- | --- | --- | --- | --- |
| AUSTRIA | Forensic | 145 | Justizanstalt Goellersdorf, Lower Austria, Goellersdorf | Alexander Dvorak |
|  | General psychiatry | NA | Social Psychiatry Center of Caritas, Vienna | Thomas Wochele |
|  | General psychiatry | NA | Division of Social Psychiatry, Medical University of Vienna | Johannes Wancata |
|  | Forensic | 53 | Klinik für Psychiatrie mit forensischem Schwerpunkt, Upper Austria, Linz | Adelheid Kastner |
| GERMANY | Forensic | 258 | Klinik für Forensische Psychiatrie und Psychotherapie, Zentrum für Psychiatrie Nordbaden, Wiesloch | Christian Oberbauer |
|  | Forensic | 185 | Klinik für Forensische Psychiatrie des Pfalzklinikums, Klingenmünster | Eva Biebinger |
|  | Forensic | 124 | Klinik für Forensische Psychiatrie und Psychotherapie, Weinsberg | Mathias C. Michel |
|  | General psychiatry | NA | Zentralinstitut für Seelische Gesundheit, Mannheim | Andreas Meyer-Lindenberg |
| ITALY | Forensic | 130 | REMS, Centro Polifunzionale ex Ospedale Stellini, Nogara | Carlo Piazza |
|  | Forensic | 20 | Sistema Polimodulare REMS, Castiglione delle Stiviere | Gianfranco Rivellini |
|  | Forensic | 20 | REMS-D, Volterra | Alfredo Sbrana |
|  | Forensic | 20 | REMS Minerva, Roma | Giuseppe Nicolò |
|  | General psychiatry | NA | IRCCS Fatebenefratelli | Giambattista Tura |
|  | General psychiatry | NA | Department of Mental Health, ASST Brescia | Antonio Vita |
| POLAND | Forensic | 60 | Institute of Psychiatry and Neurology, Department of Forensic Psychiatry, | Janusz Heitzman |
|  | Forensic | 40 | Psychiatric Hospital Starogard Gdański, Forensic Department | Agnieszka Welento-Nowacka |
|  | Controls | NA | Departments of General Psychiatry, Institute of Psychiatry and Neurology, Warsaw. | Janusz Heitzman |
| UNITED KINGDOM | Forensic and General psychiatry | 130 | South London and Maudsley NHS Foundation Trust | Marco Picchioni |
|  | Forensic and General psychiatry | 75 | South West London and St. Georges Mental Health NHS Trust | Olumuyiwa Olumoroti |
|  | General psychiatry | NA | Barnet, Enfield, Haringey Mental Health NHS Trust | Sarah Hewitt |
|  | General psychiatry | NA | Oxleas NHS Foundation Trust | Peter Stevens |
|  | General psychiatry | NA | Oxford Health NHS Foundation Trust | Duncan Dudley-Hicks |
|  | Forensic | 240 | St. Andrew’s Healthcare | Katina Anagnostakis |

*The number of beds applies only to forensic facilities, since controls have been recruited both in residential facilities and in outpatient settings
